# Digital Vasculitis in a Patient with Rheumatoid Arthritis Responded Well to Adalimumab

**DOI:** 10.1155/2014/416825

**Published:** 2014-07-15

**Authors:** Senol Kobak, Hatice Yilmaz, Murat Yalcin, Ahmet Karaarslan

**Affiliations:** ^1^Department of Rheumatology, Faculty of Medicine, Sifa University, 35100 Bornova, Izmir, Turkey; ^2^Department of Orthopedics, Faculty of Medicine, Sifa University, Turkey

## Abstract

42-year-old old female patient, followed up with diagnosis of rheumatoid arthritis for 15 years, was admitted with necrotising ulcer of left hand 1st and 2nd fingertips and pain, swelling, limitation of movement, and morning stiffness at bilateral wrist, and metacarpophalangeal and proximal interphalangeal joints. Laboratory tests revealed elevated acute phase reactants. Radial and ulnar arteries were clear in upper extremity Doppler ultrasound. The patient was diagnosed as RA activation and digital ulcer and administered iloprost infusion for five days and 1 mg/kg corticosteroid and 20 mg/week methotrexate (MTX). After one month, a partial regression of clinical and laboratory findings was observed. However, 6 months later, due to relapsed and increased complaints and findings, adalimumab 40 mg was administered. Two months later, clinical and laboratory findings apparently decreased.

## 1. Introduction

Rheumatoid arthritis (RA) is an autoimmune, chronic, systemic inflammatory disease with unknown etiology and symmetric arthritis. Most commonly, the disease not only affects wrist and metacarpophalangeal (MCP) and proximal interphalangeal (PIP) joints but can also affect larger joints like shoulder, elbow, and knee. Although musculoskeletal system involvement is dominant, it can also cause extra-articular involvement as lung, heart, or vasculitis. Rheumatoid vasculitis (RV) is a rare complication of RA patients [1]. RA-related vasculitis can cause different clinical findings like isolated digital infarcts or systemic involvement. Most commonly, it causes ulcerated lesions around medial and lateral malleolus in lower extremities with necrotizing vasculitis in median size vessels. RV is more frequent in the patients with severe joint involvement, longer disease duration, rheumatoid factor (RF), and anti-CCP positivity [2]. Leg ulcers, digital infarcts, scleritis, mononeuritis multiplex, and pauci-immune glomerulonephritis can be present in RV [3, 4]. Digital ulcer is a complication of Raynaud phenomenon which is commonly seen in scleroderma and SLE patients, caused by episodic digital ischemia [5]. It is very rare in RA patients. In this case report, isolated digital ulcer is detected in a seronegative RA patient and partially treated with prostacyclin analogue, methotrexate (MTX), and corticosteroid. Significant regression was observed with adalimumab treatment.

## 2. Case Report

**Figure 1:**
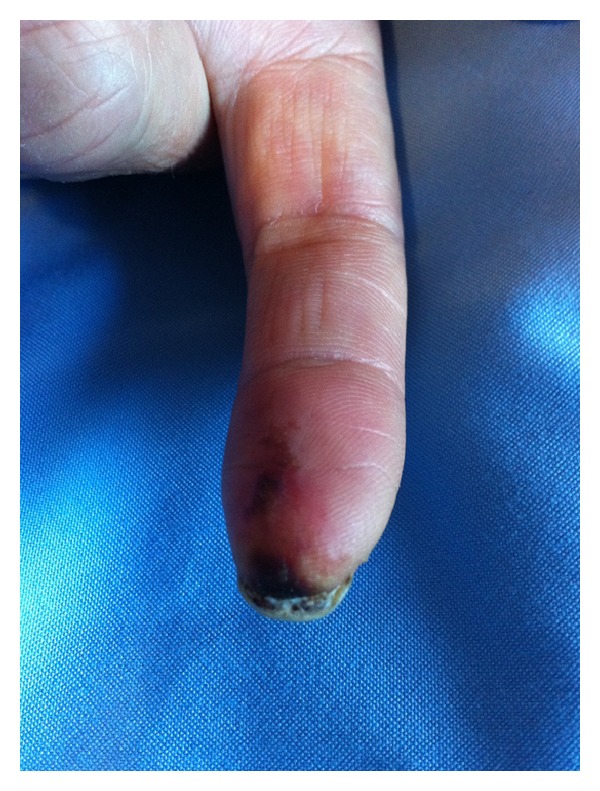
Left hand 2nd fingertip with vasculitic necrotising lesion.

**Figure 2:**
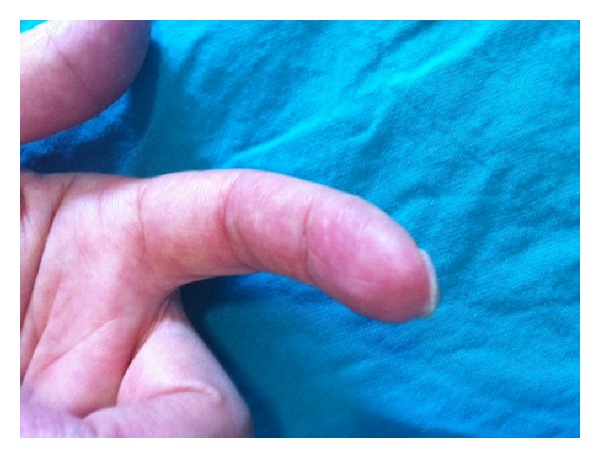
Left hand 2nd fingertip after adalimumab treatment.

42-year-old female patient, followed up with diagnosis of rheumatoid arthritis for 15 years, was admitted at our rheumatology clinic with necrotizing ulcer of left hand 1st and 2nd fingertips and pain, swelling, limitation of movement, and morning stiffness at bilateral wrist and metacarpophalangeal and proximal interphalangeal joints. According to her medical history she left her medications (MTX and low dose corticosteroid) two years ago without any polyclinic control visits. She has never used drugs such as ergotamine/caffeine. Examination revealed tenderness, swelling, limitation of movement in bilateral wrist and MCP and PIP joints, and necrotising, painful, ulcerated lesions in left hand 1st and 2nd fingertips (Figure 1). Laboratory tests revealed C-reactive protein (CRP): 2.9 mg/dL (normal 0–0.5) and erythrocyte sedimentation rate (ESR): 57 mm/h (normal ≤20 mm/h). Liver enzymes and kidney functions were normal; hemogram revealed chronic disease anemia, mild thrombocytosis, and leukocytosis. Serological tests were performed; cryoglobulin, ANCA, rheumatoid factor (RF), anti-cyclic citrullinated peptide (CCP) antibody, and anti-nuclear antibody (ANA) were negative. Radial and ulnar arteries were clear in upper extremity Doppler ultrasound in the level of wrist. Thorax X-ray, pelvic ultrasound, and echocardiography were normal. Hand and wrist X-ray showed radiologic signs consistent with RA. Upper extremity capillaroscopy revealed vascular disease; there were no signs of scleroderma. Clinical and radiographic findings taken together, RA activation and digital vasculitis were diagnosed. The patient was administered iloprost trometamol infusion for five days, and 1 mg/kg/day corticosteroid, 20 mg per week methotrexate, and aspirin 100 mg/day were started. After one month, clinically the lesion in the 1st finger regressed and the ulcerated lesion in the 2nd finger was much better. Six months later, the patient was readmitted to our outpatient clinic due to pain, swelling, and morning stiffness longer than 1 hour in bilateral wrist and MCP and PIP joints and relapse of ulcerated skin lesions in left hand 1st and second fingertips. Examination revealed arthritis in bilateral wrist, MCP, PIP, right knee, and tenderness in bilateral elbow and shoulder joints. Laboratory tests revealed CRP: 8.2 mg/dL, ESR: 54 mm/h, and chronic disease anemia; liver functions, kidney functions, and routine urine analysis were normal. DAS28 was >5.1. The patient was diagnosed as treatment resistant active rheumatoid arthritis and adalimumab 40 mg/once 14 days sc was started. In the second month of treatment, she was much better in her complaints, in her examination arthritis had disappeared, and the ulcerated lesions in her left hand fingertips had been resolved (Figure 2). Laboratory tests revealed normal acute phase reactants. Her treatment and follow-up are going on and she has no complaints meanwhile.

## 3. Discussion

Rheumatoid vasculitis (RV) is a rare complication of RA. Skin involvement is present in more than 90% of patients; it is usually characterized by deep skin ulcers over lateral and medial malleolus. Pathogenesis of RA is not yet clear, but immune system dysregulation and humoral immunity may have a role. In a study, antibodies against nuclear soluble proteins (ENA) are thought to be an indicator of circulating immune complexes and their relationship with RA systemic complications and extra-articular findings. In particular in rheumatoid vasculitis, they make complexes with ENA in the vessel wall and present as free antibody in circulation [6]. In a patient with RA and Felty syndrome, digital vasculitis after splenectomy was reported [7]. In another study, in a RA patient with skin necrosis and digital infarcts cryofibrinogenemia and high cryofibrinogen were detected and this was thought to worsen necrotising skin lesions [8]. Corticosteroids and drugs with different mode of action (methotrexate, cyclophosphamide, and azathioprine) are used in the treatment of RV. Literature about the usage of anti-TNF-alpha drugs in RV is controversial. There is data about their efficacy, but on the other hand vasculitis may occur. A patient with small vessel vasculitis had given response to anti-TNF*α* [9]. In different studies, patients with RV and mononeuritis multiplex and drop foot, efficacy and safety of infliximab were proven [10, 11]. Besides, vasculitis due to anti-TNF*α* agents was reported in literature. In a review, 113 of 233 autoimmune disorder patients had vasculitis due to anti-TNF-*α* use [12]. According to this review, 59 of the patients who had vasculitis were using etanercept (52%), 47 were using infliximab (42%), and 5 were using adalimumab (4%). Infliximab using patients had visceral vasculitis more often than etanercept using patients. Visceral involvement was most often as peripheral neuropathy and renal vasculitis. The primary disease of the patients who had infliximab- and etanercept-related vasculitis was RA and internal organ involvement was more often 29% and 6% [13]. The most common histopathological type of anti-TNF-*α* related vasculitis was leukocytoclastic vasculitis and the most common area of involvement was skin. In some of the cases, cutaneous finding may be digital vasculitis. The mechanism of vasculitis due to anti-TNF-*α* drugs is unknown. TNF-*α* and anti-TNF-*α* may produce immune complex and accumulate in the small capillary vessel wall according to one theory. This accumulation may cause type III hypersensitivity reaction and vasculitis. According to another theory, anti-TNF-*α* drugs may increase autoantibody formatting and cause cytokine imbalance. These changes can cause lupus-like reactions and vasculitis [14, 15]. Because of the limited data, we cannot know how different anti-TNF-*α* agents cause vasculitis or how to change anti-TNF-*α* treatment in a patient with vasculitis. Further studies are necessary in this subject.

In conclusion we report a patient with seronegative RA and digital vasculitis responded well to adalimumab therapy. Consequently, digital vasculitis is a rare condition in RA, but we proved that early diagnosis and proper treatment may cause pleasant results. Anti-TNF-alfa drugs should be considered in treatment. Because of the conflicting results between these drugs and vasculitis, more studies with more patient numbers are necessary.
